# Risk indicators for venous thrombosis in first-degree relatives of patients with recurrent venous thromboembolism in Chinese

**DOI:** 10.1097/MD.0000000000004539

**Published:** 2016-10-14

**Authors:** Lianxing Zhao, Chunsheng Li, Rui Shao, Yingying Fang

**Affiliations:** Department of Emergency Medicine, Beijing Chao-Yang Hospital, Capital Medical University, Chao-Yang District, Beijing, China.

**Keywords:** endothelial nitric oxide synthase, family, recurrence, single-nucleotide polymorphism, venous thrombosis

## Abstract

Having encountered several subjects with venous thromboembolism (VTE) in 1 family in which 1 proband has recurrent VTE (rVTE), we aimed to assess the risk of VTE in first-degree relatives, especially the children of individuals with rVTE, and to investigate the association of endothelial nitric oxide synthase (eNOS) G894T polymorphism between Chinese persons with rVTE and their offspring.

We collected information about family histories and blood samples from 126 individuals with rVTE who had presented to our institute from 2003 to 2014, and 126 population-based controls and the first-degree relatives of subjects in these 2 groups. We tested blood samples for heritable thrombophilia and calculated odds ratios (ORs) and kappa coefficients.

First-degree relatives of individuals with rVTE patients had a statistically significant risk of developing VTE (OR 2.62, 95% confidence interval [CI] 1.61–4.26, *P* < 0.001). For siblings, the OR was 2.72 (95% CI 1.56–4.73, *P* < .001). Moreover, for each year that the patient was older, the OR was 0.98 (95% CI 0.97–0.99, *P* = 0.03). One (11.2%) of the 9 individuals who had the same eNOS G894T polymorphism as their probands had a history of VTE, whereas none of the 17 relatives without the same polymorphism had developed VTE. The associations between patients and their children were statistically significant for VTE (kappa = 0.23, *P* < 0.001) and for eNOS G894T (kappa = 0.03, *P* = 0.04).

In this case-controlled study, we demonstrated a higher risk of VTE among first-degree relatives of individuals with rVTE, especially in siblings of younger subjects with rVTE. We also found that eNOS G894T polymorphism may be a predictor of VTE in offspring of individuals with rVTE.

## 1 Introduction

Venous thromboembolism (VTE), a multifactorial disease, involves an interaction between hereditary and acquired risk factors,^[1,2]^ and is the third most common cardiovascular disease after ischemic heart disease and stroke.^[3]^ Deep venous thrombosis (DVT), one of the presentations of VTE, has significant morbidity and pulmonary embolism (PE) is a life-threatening disease with a short-term survival of less than 60%.^[4]^

During the past decade, many genetic risk factors have been found to be associated with VTE, with these factors varying among different regions and races.^[5]^ Factor V Leiden^[6]^ and the G202210A prothrombin gene^[7]^ are the most common susceptibility alleles in Caucasians, whereas in Chinese individuals, inherited deficiency of antithrombin, protein C, and protein S may play important roles in the occurrence of VTE.^[8–11]^ In addition, nitric oxide (NO) produced by endothelial NO synthase (eNOS) has both vasodilatory and antithrombotic properties. In a previously published meta-analysis, we found that eNOS G894T polymorphism may be a risk factor for venous thrombosis and may increase the risk of developing thrombotic disease in Asia.^[12]^ In 1905, Briggs^[13]^ was the first to report clustering of venous thrombophlebitis in a family. Evidence of familial aggregation is 1 avenue for studying the role of genetics in this disease. Several authors^[14–16]^ have studied family history as a risk factor for VTE and the risk of VTE in first-degree relatives of patients with their first VTE.^[17]^ However, few have studied the risk of VTE in first-degree relatives of patients with thrombophilia and recurrent VTE (rVTE).

The current study was designed to estimate more accurately the risk of VTE in first-degree relatives of individuals with rVTE and to study the association of eNOS G894T polymorphism between Chinese patients and their first-degree relatives.

## 2 Methods

### 2.1 Study subjects

Individuals with rVTE who had been admitted to Beijing Chao-Yang Hospital, Capital Medical University, and Beijing An Zhen Hospital, Capital Medical University, between January 28, 2003 and August 23, 2014 were enrolled in this study. DVTs were diagnosed by color duplex ultrasound scanning and PE by high probability on ventilation-perfusion lung scans or spiral computed tomography (CT) pulmonary angiography. Healthy volunteers who had visited the Center for Health Checks of Beijing Chao-Yang Hospital, Capital Medical University, between September 20, 2012 and June 15, 2014 were also enrolled. No control subjects had any evidence or history of VTE or had received anticoagulant therapy. Control subjects were matched by age (±2 years) and sex with the patient group. Blood samples were obtained from all probands (both patients and controls) and at least 1 of the offspring in each family at least 3 months after discontinuation of vitamin K antagonist therapy. Recurrent DVT was defined as diagnosis of an acute thrombus in a previously uninvolved segment of vein or an increase of more than 4 mm in the size of the residual material on color duplex ultrasound scanning.^[18,19]^ Recurrent PE was defined as finding a new intraluminal filling defect on CT pulmonary angiography or a new perfusion defect in a segment with normal ventilation.^[20]^

Probands and first-degree relatives had to be at least 18 years old to be eligible. Data on first-degree relatives who had died could be included with the consent of related living study subjects. This study was approved by the Institutional Review Board of Beijing Chao-Yang Hospital, Capital Medical University, and was performed in accordance with the ethical standards as laid down in the 1964 Declaration of Helsinki and its later amendments or comparable ethical standards. All case and control subjects gave their written informed consent for participation in the study and for DNA analysis.

Diagnosis of rVTE required the absence of acquired risk factors such as injury, immobilization, or plaster cast for at least 1 week in the previous 3 months, diagnosis of malignancy within the previous 5 years, and receiving hormone therapy on the index date of VTE occurrence.

### 2.2 Clinical data

Demographic data and general characteristics of case and control probands were collected by one-to-one standardized interviewing using the same questionnaire. The family history of probands included the number of first-degree relatives (parents, siblings, and offspring) and the number of first-degree relatives with a history of VTE.

A standard questionnaire was also used to collect data on first-degree relatives of cases and controls. Demographic data, general characteristics, history of VTE, age at diagnosis of VTE, category of relative, and type of VTE were obtained from probands and checked with those relatives by face-to-face or telephone interviews.

### 2.3 Laboratory analysis

Venous blood samples were collected from participants into vacutainer tubes containing 1/10 volume of 0.105 mol/L trisodium citrate. After centrifugation at 2000*g* for 20 minutes, plasma was used for coagulation assays and white blood cells for extracting DNA.

Activities of protein C, protein S, and antithrombin were measured on a Sysmex CA 7000 Analyzer (Sysmex, Japan) using reagents from Siemens Healthcare Diagnostics Products (GmbH, Germany). Protein C and antithrombin activities were assayed by a chromogenic substrate method and activity of protein S by a clotting method based on prothrombin time. Deficiency was diagnosed only in cases where the activity of antithrombin, protein C, or protein S was below the lower limit of the normal range, and both liver and renal functions were normal for Chinese subjects.

The genotype for eNOS G894T polymorphism, which contributes the amino acid residues from Glu to Asp in code 298, was determined. Genomic DNA was extracted from white blood cells using a DNA extraction kit (RelaxGene Blood DNA System, TianGen, China). Oligonucleotide primers 5′-CAC GGA GAC CCA GCC AAT G-3′ (forward) and 5′-CCC ACC CAG TCA ATC CCT TT-3′ (reverse) were designed using Primer Premier 5.0.

Amplification by PCR was performed in a volume of 50 μL containing <1 μg of genomic DNA, 1 μL of each 10 μM primer, 5 μL of 10× Pyrococcus furiosis buffer, 4 μL of 2.5 mM deoxy-ribonucleoside triphosphate mixture, and 0.8 μL of 2.5 U/μL Taq Plus DNA polymerase (TianGen, China) dilute in ddH_2_O. PCR conditions were initial denaturation stage at 94°C for 3 minutes, followed by 30 cycles of 94°C for 30 seconds, 63°C for 30 seconds, 72°C for 1 minute, and a final extension at 72°C for 5 minutes. The product of 287 bp was directly sequenced and digested by MboI (New England Biolabs) for 16 hours at 37°C to be cleaved into 189 and 98 bp at nucleotide 894 if there was a T. All products were resolved by electrophoresis on a 2% agarose gel stained with GenGreen nucleic acid gel stain for analysis.

### 2.4 Statistical analysis

Normally distributed quantitative data are presented as mean (SD) and those distributed non-normally as median (25th and 75th); qualitative data are presented as frequency (percentage). Odds ratios (ORs) and 95% confidence intervals (CIs) were calculated to assess associations between risk of VTE in first-degree relatives and characteristics of probands with rVTE. Logistic regression by the method of generalized estimating equations, which accounts for within-family correlations among relatives, was used to compute the ORs and 95% CIs. Student *t* test was used for continuous data and the chi-square or Fisher exact test was used to compare genotypic frequencies of eNOS G894T between probands and between their offspring. Prediction of family history was calculated by a diagnostic test for identifying genetic risk (eNOS G894T). The kappa coefficient was calculated to estimate the association between probands and their offspring. A *P* value of <0.05 was considered to indicate significant difference. All statistical analyses were performed with SPSS 19.0 (SPSS, Chicago, IL).

## 3 Results

### 3.1 Characteristics of the study subjects

**Figure 1:**
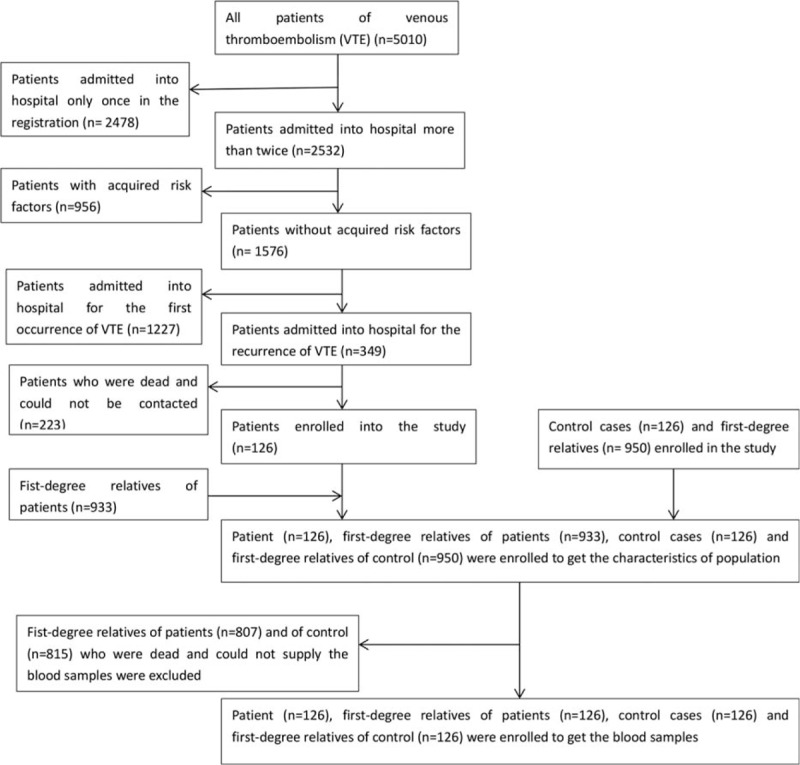
The flow chart of enrolling probands and their first-degree relatives.

Between January 28, 2003 and August 23, 2014, 126 patients with rVTE were eligible to be enrolled in this study. Between September 20, 2012 and June 15, 2014, 126 healthy people attending a Center for Health Checks were selected to be controls (Fig. 1). As shown in Table 1, 60.3% of case probands and 61.9% of control probands were female. Case and control probands were of similar age. The age range of the case probands was 21 to 90 years (mean ± SD, 62.8 ± 14.9 years) and of the control probands 23 to 91 years (mean ± SD, 63.2 ± 13.5 years). DVT and PE, which account for most VTE, were the diagnoses in 73.8% of the cases.

**Table 1:**
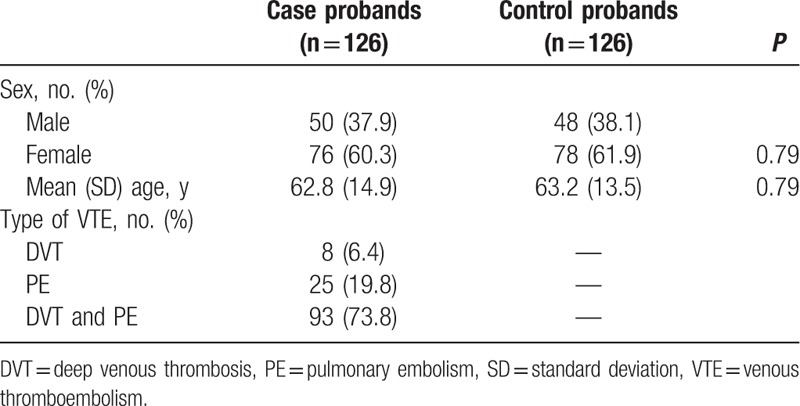
Characteristics of case and control probands.

The study subjects also included 933 first-degree relatives of cases and 950 first-degree relatives of controls. As shown in Table 2, the sex distribution was similar for case relatives and control relatives, with 51.1% of case relatives and 50.6% of control relatives being female; this difference is not statistically significant (*P* = 0.97) in the distribution of types of relatives. The mean (SD) number of first-degree relatives assessed (in addition to the probands) was 7.4 (1.8) in case families and 7.5 (1.9) in control families (*P* = 0.30). The number of siblings in case families (mean ± SD, 3.1 ± 1.4) was not significantly different from that in control families (mean ± SD, 3.1 ± 1.3) (*P* = 0.47), nor was the number of offspring (for case probands: mean ± SD, 2.3 ± 1.1; for control probands: mean ± SD, 2.4 ± 1.1) (*P* = 0.51). Sixty-one case relatives and 24 control relatives reported having had VTE.

**Table 2:**
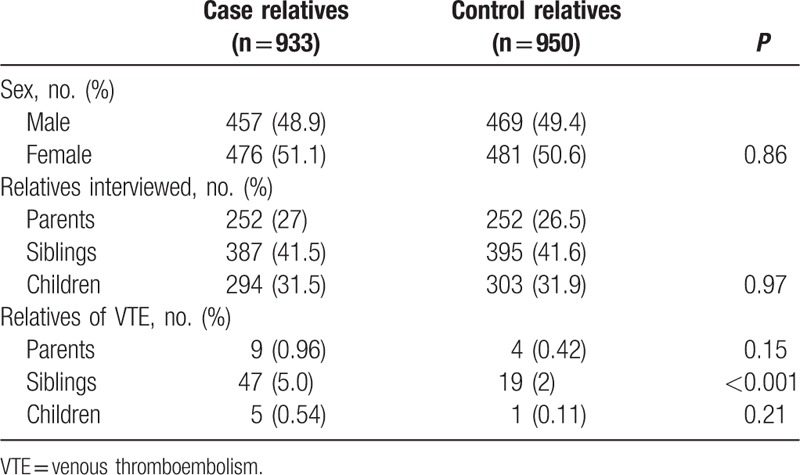
Characteristics of case and control first-degree relatives.

### 3.2 Association between history of VTE in probands and VTE risk in first-degree relatives

More case relatives than control relatives had been diagnosed with VTE (6.5% vs 2.5%) (Table 2). As shown in Table 3, according to a multivariable model (including age, sex, and family history of rVTE), the association between diagnosis of VTE in first-degree relatives and a family history of rVTE was statistically significant (OR 2.62, 95% CI 1.61–4.26, *P* < 0.001). This was especially true for siblings (OR 2.72, 95% CI 1.56–4.73, *P* < 0.001), whereas the OR was 2.09 (95% CI 0.64–6.82, *P* = 0.22) and 4.97 (95% CI 0.57–43.15, *P* = 0.15) for parents and offspring, respectively.

**Table 3:**
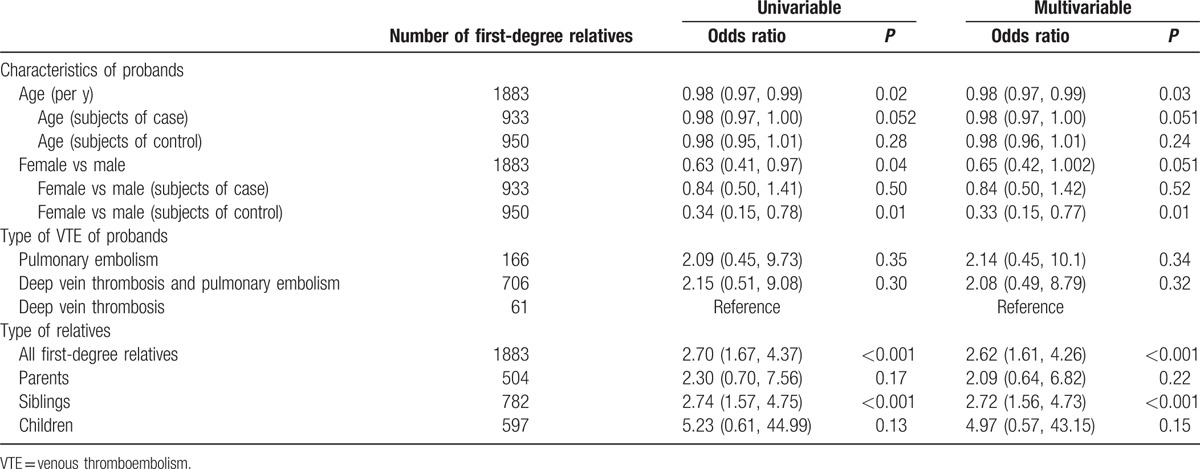
Predictors of VTE in first-degree relatives.

### 3.3 Association between characteristics of probands and VTE risk in first-degree relatives

As shown in Table 3, there was an inverse relationship between the risk of VTE in first-degree relatives and the age of probands. For each additional year of age of a proband, the OR was 0.98 (95% CI 0.97–0.99, *P* = 0.03) for all subjects; it was 0.98 (95% CI 0.97–1.00, *P* = 0.051) for subjects in the case group. The sex of the proband was associated with a higher risk of VTE in first-degree relatives for all subjects according to univariable analysis (female vs male: OR 0.63, 95% CI 0.41–0.97, *P* = 0.04); however, this association was no longer statistically significant according to multivariable analysis (female vs male: OR 0.65, 95% CI 0.42–1.002, *P* = 0.051); for subjects in the control group, ORs were statistically significant according to both univariable (OR 0.34, 95% CI 0.15–0.78, *P* = 0.01) and multivariable analysis (OR 0.33, 95% CI 0.15–0.77, *P* = 0.01). The type of VTE of proband was not predictive of thrombosis in the first-degree relatives of subjects with rVTE.

### 3.4 Association of genetic factors between case and control probands and their first-degree relatives

As shown in Table 4, there was a significant difference between case probands and control probands, so was between case relatives and control relatives for various genetic factors (including protein C, protein S, and eNOS G894T, but not antithrombin). When 26 relatives of the 26 cases with rVTE who had eNOS G894T polymorphism were tested for the same gene abnormality, it was found that 1 (11.2%) of the 9 relatives who did have the eNOS G894T polymorphism had a history of VTE, whereas none of the 17 relatives without this polymorphism had developed VTE. However, the area under the receiver-operating characteristic curve for family history of rVTE and identified eNOS polymorphism was only 50.3%, which indicates a pool association between them.

**Table 4:**
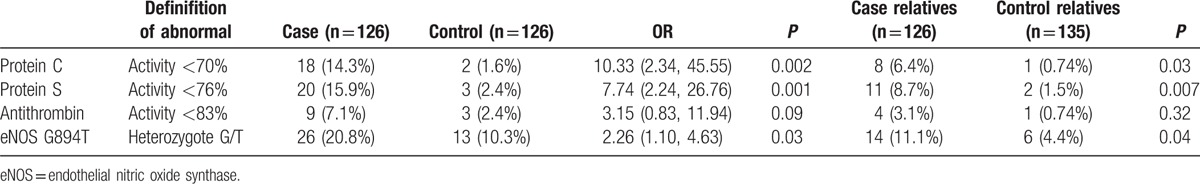
Genetic risk factors of probands and first-degree relatives.

### 3.5 Association of VTE and eNOS G894T polymorphism between probands and offspring

Blood samples were collected from 1 offspring of every proband (both case and control group). The kappa coefficients for VTE and eNOS G894T between probands and their offspring in the 2 groups were calculated. As shown in Table 5, there was a statistically significant association in the case group (for VTE: kappa = 0.23, *P* < 0.001; for eNOS G894T: kappa = 0.03, *P* = 0.04); however, the correlation for eNOS G894T was poor. The correlation was not statistically significant in the control group (for VTE: kappa = 0.001, *P* = 0.52; for eNOS G894T polymorphism: kappa = 0.01, *P* = 0.10).

**Table 5:**
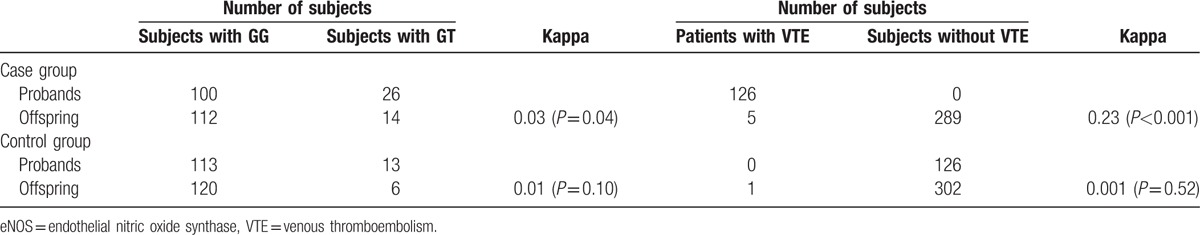
Relationship of eNOS G894T polymorphism in probands and their offspring.

## 4 Discussion

This case-control family clustering study aimed to assess associations between thrombophilic subjects with rVTE and their first-degree relatives. First, we found that the risk of VTE in the first-degree relatives of individuals with rVTE is 2.62-fold that in healthy controls (*P* < 0.001). This association is even more pronounced in siblings of subjects with rVTE (OR 2.72, *P* < 0.001). Most of the study subjects’ parents had died; their histories were obtained from their relatives. A response of “I don’t know” to a question about a parent's history was taken to indicate a negative VTE history; this may have contributed to the lack of statistical significance. Additionally, most offspring were too young to have developed VTE at the time of the study, which may also have contributed to the lack of statistical significance. Second, we demonstrated that the younger a case proband was at diagnosis, the higher the risk of VTE in their first-degree relatives. For each additional year of age of a proband, the OR was 0.98. These 2 factors would have affected the risk of VTE in first-degree relatives independently of the genetic factors in the case group. Third, the sex of a proband with rVTE also likely influences the risk for first-degree relatives, with the OR being 0.65 for female versus male for all subjects (borderline *P* value, *P* = 0.051); this influence was clearly significant in the control group (OR 0.33, *P* = 0.01). In our cohort, 43.5% of male subjects and 34.2% of female subjects had relatives with VTE, indicating the importance of sex in heritance of VTE. Fourth, the ability to predict occurrence of VTE in affected subjects’ relatives is likely greater in those with the same genetic abnormality (eNOS G894T) as the corresponding probands. Fifth, there was a statistically significant association between occurrence of VTE and carrying eNOS G894T polymorphism in Chinese offspring and their parents in the case group.

Some studies of the association between family history and risk of VTE in relatives of patients have been published^[14–17,21]^; however, the patients in those studies had experienced only 1 episode of VTE, whereas our patients had rVTE, that is, thrombophilia. Additionally, we studied the influence of proband characteristics on their first-degree relatives rather than the effects of history of first-degree relatives on probands. The genetic factors for VTE vary among different regions and races.^[5]^ We are not aware of previous studies that have explored the risk of VTE in first-degree relatives of Chinese subjects.

The strengths of our study include the case probands’ history of rVTE and our assessment of genetic factors associated with VTE. We also demonstrated that family history is an independent risk factor for occurrence of VTE and could therefore be added to a prior VTE history to diagnose clinical thrombophilia that is not necessarily related to carrying a known inheritable risk factor.^[15]^ Although antithrombin, protein C, and protein S^[8,22,23]^ are important risk factors in Chinese patients, investigators have found no preponderant mutation.^[24,25]^ Many mutations in the genes encoding these factors can cause deficiency; the activities of the corresponding proteins serve as surrogates for assessing genetic defects. Recent studies^[26–28]^ have demonstrated the relationship of eNOS G894T polymorphism with venous thrombosis, especially in Chinese subjects. We explored this genetic factor in patients with rVTE and their first-degree relatives.

Our study has several limitations. First, not all the interviews in the study were face to face; some were telephonic interviews. Because most parents had died, information about them was obtained only by asking their relatives. Second, the sample size was small, including only patients with records of rVTE. Some patients who had been treated as outpatients and not registered were not included in our study. The small sample size prevents drawing any firm conclusions, especially concerning the value of eNOS G894T polymorphism in predicting VTE risk among relatives of VTE patients: only 1 of 9 relatives with eNOS G894T polymorphism had a history of VTE. A larger sample may have resulted in more significantly statistical outcomes. Third, thrombosis is a multifactorial disease; the only heritable factors we evaluated were antithrombin, protein C, protein S, and eNOS G894T. Fourth, for the analysis of associations between offspring and parents, we did not collect blood samples from all offspring.

Our study has important clinical implications for relatives of individuals with rVTE. First, all such persons should inform their first-degree relatives, especially their siblings, that they have a higher risk than normal of developing thrombosis and should therefore get prophylaxis when in high-risk situations. Although we identified only a weak correlation with eNOS G894T, which demonstrates that VTE is a multifactorial disease involving several genetic risk factors, the association of VTE occurrence between parents and offspring was relatively strong. Second, genetic causes should be further studied; we have not yet identified the predominant factor associated with VTE. Relatives with the same genetic factors as their proband have a higher risk of developing VTE.

## 5 Conclusions

In conclusion, this study revealed a higher risk of VTE in first-degree relatives of rVTE patients, especially in siblings of younger patients, and also found that eNOS G894T polymorphism may be a predictor of VTE in offspring of individuals with rVTE.

## Acknowledgment

The authors thank YuHong Mi, Beijing An Zhen Hospital, Capital Medical University for helping us to contact patients.

## References

[1] Rosendaal FR Venous thrombosis: a multicausal disease. *Lancet* 1999; 353:1167–1173. 10209995 10.1016/s0140-6736(98)10266-0

[2] Anderson FA Jr Spencer FA Risk factors for venous thromboembolism. *Circulation* 2003; 107 (23 suppl 1):I9–I16. 12814980 10.1161/01.CIR.0000078469.07362.E6

[3] Kniffin WD Jr Baron JA Barrett J The epidemiology of diagnosed pulmonary embolism and deep venous thrombosis in the elderly. *Arch Internal Med* 1994; 154:861–866. 8154949

[4] Silverstein MD Heit JA Mohr DN Trends in the incidence of deep vein thrombosis and pulmonary embolism: a 25-year population-based study. *Arch Internal Med* 1998; 158:585–593. 9521222 10.1001/archinte.158.6.585

[5] Margaglione M Grandone E Population genetics of venous thromboembolism. A narrative review. *Thromb Haemost* 2011; 105:221–231. 20941456 10.1160/TH10-08-0510

[6] Rees DC The population genetics of factor V Leiden (Arg506Gln). *Br J Haematol* 1996; 95:579–586. 8982030 10.1046/j.1365-2141.1996.d01-1954.x

[7] Rosendaal FR Doggen CJ Zivelin A Geographic distribution of the 20210 G to A prothrombin variant. *Thromb Haemost* 1998; 79:706–708. 9569177

[8] Zhu T Ding Q Bai X Normal ranges and genetic variants of antithrombin, protein C and protein S in the general Chinese population. Results of the Chinese Hemostasis Investigation on Natural Anticoagulants Study I Group. *Haematologica* 2011; 96:1033–1040. 21486865 10.3324/haematol.2010.037515 PMC3128223

[9] Gu Y Shen W Zhang L Deficiency of antithrombin and protein C gene in 202 Chinese venous thromboembolism patients. *Int J Lab Hematol* 2014; 36:151–155. 24028705 10.1111/ijlh.12146

[10] Tang L Jian XR Hamasaki N Molecular basis of protein S deficiency in China. *Am J Hematol* 2013; 88:899–905. 23813890 10.1002/ajh.23525

[11] Ding Q Wang M Xu G Molecular basis and thrombotic manifestations of antithrombin deficiency in 15 unrelated Chinese patients. *Thromb Res* 2013; 132:367–373. 23932013 10.1016/j.thromres.2013.07.013

[12] Zhao L Li C Yin Q Endothelial nitric oxide synthase 894G>T polymorphism and thrombotic disease: a Meta-Analysis of 17 studies involving 8808 subjects. *Thromb Res* 2014; 134:1057–1065. 25081832 10.1016/j.thromres.2014.07.014

[13] Briggs JB Recurring phlebitis of obscure origins. *Johns Hopkins Hosp Bull* 1905; 16:228–233.

[14] Hron G Eichinger S Weltermann A Family history for venous thromboembolism and the risk for recurrence. *Am J Med* 2006; 119:50–53. 16431184 10.1016/j.amjmed.2005.04.043

[15] Noboa S Le Gal G Lacut K Family history as a risk factor for venous thromboembolism. *Thromb Res* 2008; 122:624–629. 18281082 10.1016/j.thromres.2007.12.026

[16] Bezemer ID van der Meer FJ Eikenboom JC The value of family history as a risk indicator for venous thrombosis. *Arch Internal Med* 2009; 169:610–615. 19307525 10.1001/archinternmed.2008.589

[17] Couturaud F Leroyer C Tromeur C Factors that predict thrombosis in relatives of patients with venous thromboembolism. *Blood* 2014; 124:2124–2130. 25049279 10.1182/blood-2014-03-559757 PMC4186541

[18] Tan M van Rooden CJ Westerbeek RE Diagnostic management of clinically suspected acute deep vein thrombosis. *Br J Haematol* 2009; 146:347–360. 19466972 10.1111/j.1365-2141.2009.07732.x

[19] Needleman L Update on the lower extremity venous ultrasonography examination. *Radiol Clin North Am* 2014; 52:1359–1374. 25444111 10.1016/j.rcl.2014.08.001

[20] Olie V Zhu T Martinez I Sex-specific risk factors for recurrent venous thromboembolism. *Thromb Res* 2012; 130:16–20. 22100316 10.1016/j.thromres.2011.10.026

[21] Couturaud F Leroyer C Julian JA Factors that predict risk of thrombosis in relatives of patients with unprovoked venous thromboembolism. *Chest* 2009; 136:1537–1545. 19592474 10.1378/chest.09-0757

[22] Ding Q Shen W Ye X Clinical and genetic features of protein C deficiency in 23 unrelated Chinese patients. *Blood Cells Molecules Dis* 2013; 50:53–58. 10.1016/j.bcmd.2012.08.004 22951146

[23] Tang L Guo T Yang R Genetic background analysis of protein C deficiency demonstrates a recurrent mutation associated with venous thrombosis in Chinese population. *PloS One* 2012; 7:e35773. 22545135 10.1371/journal.pone.0035773 PMC3335791

[24] Luxembourg B Delev D Geisen C Molecular basis of antithrombin deficiency. *Thromb Haemost* 2011; 105:635–646. 21264449 10.1160/TH10-08-0538

[25] Tang L Wang HF Lu X Common genetic risk factors for venous thrombosis in the Chinese population. *Am J Human Genet* 2013; 92:177–187. 23332921 10.1016/j.ajhg.2012.12.013 PMC3567266

[26] Li YY Zhai ZG Yang YH Association of the 894G>T polymorphism in the endothelial nitric oxide synthase gene with risk of venous thromboembolism in Chinese population. *Thromb Res* 2011; 127:324–327. 21320716 10.1016/j.thromres.2010.11.034

[27] Akhter MS Biswas A Ranjan R The nitric oxide synthase 3 gene polymorphisms and their association with deep vein thrombosis in Asian Indian patients. *Clin Chim Acta* 2010; 411:649–652. 20114041 10.1016/j.cca.2010.01.025

[28] Heil SG Den Heijer M Van Der Rijt-Pisa BJ The 894>T variant of endothelial nitric oxide synthase (eNOS) increases the risk of recurrent venous thrombosis through interaction with elevated homocysteine levels. *J Thromb Haemost* 2004; 2:750–753. 15099281 10.1111/j.1538-7836.2004.00701.x

